# Comparison of cancer and all-cause death rates of Australian rock and pop musicians, footballers, cricketers and the general population

**DOI:** 10.1016/j.jsampl.2024.100070

**Published:** 2024-07-11

**Authors:** John W. Orchard, Tim Driscoll, Angus Davis, Elizabeth Driscoll, Jessica J. Orchard

**Affiliations:** a School of Public Health, University of Sydney, Australia; b The Kinghorn Cancer Centre at St Vincent's Hospital (Sydney), Australia

**Keywords:** Exercise, Standardized mortality ratio, Musicians, Longevity

## Abstract

**Introduction:**

Multiple international studies, including some from Australia, have found that professional and elite athletes have lower death rates/greater life expectancy than the general population. By contrast, international rock and pop musicians have been found to die prematurely compared to the general population, although no studies have focused on Australian musicians specifically.

**Methods:**

Death rates and life expectancy were compared for cohorts of Australian rock and pop musicians, male VFL/AFL footballers and male Sheffield Shield cricketers and the general Australian population, for the years 1971–2022 inclusive. Data were sourced from Wikipedia.

**Results:**

A cohort of 655 Australian rock and pop musicians showed an increased Standardized Mortality Rate (SMR) of 1.35 (95% CI 1.07–1.71). The rate of cancer deaths in musicians showed an even higher SMR of 1.85 (95% CI 1.32–2.59). The male Australian football, SMR 0.77 (95% CI 0.74–0.80), and cricket, SMR 0.71 (95% CI 0.64–0.78), athlete cohorts showed significantly decreased death rates compared to the general Australian male population. Life expectancy for the male athlete groups after age 20 roughly tracked the superior life expectancy of the general Australian female population, whereas life expectancy for the male musician group was slightly inferior to the general Australian male population.

**Discussion:**

The likely increase in cancer deaths in Australian musicians could be explained by substance exposure (alcohol, tobacco, both active and passive, and perhaps other illicit substances), although this causation was not specifically assessed as part of our study. Unlike other international musician cohorts, we did not observe a high rate of deaths directly due to drug overdose in the 3rd and 4th decades of life. The likely explanation for the better health of the athlete cohorts is the known survival benefit of high levels of exercise (primarily through reduced cardiovascular disease and cancers).

## 1 Introduction

Multiple studies from different sports have shown that elite athletes outlive the general population. This has been shown by systematic review, and particularly in Olympic athletes [[1], [2], [3]]. There have also been recent studies showing the same trend in the football codes [4,5]. With respect to Australian athletes, Olympians (of both sexes) [6], male AFL footballers [5] and male cricketers [7] all outlive the general population. It is difficult to differentiate between a health benefit of playing professional/elite sport and what is called the “Healthy worker hire effect” (HWHE). For professional athletes to debut in elite sport, they must be relatively free of major disease/illness/injury at a young age, whereas the general population will contain a (small) percentage of people in their late teens or early 20s who have chronic illness or injury that could shorten their life expectancy. It is understood the HWHE exists but it is very hard to estimate its contribution.

A somewhat similar celebrity group to athletes is popular musicians. Musicians (in the rock and pop genres) start their careers in a very similar fashion to athletes. They typically would become well-known in their field in their early 20s, and are probably also subject to the HWHE when they start performing and recording professionally. They also have similar life benefits and challenges to athletes, in that they have the benefit of earning a high income at a young age, but the challenge that this income may drop in middle age and also the challenges associated with the pressure of being publicly well-known or famous. A difference is that athletes tend to be outdoor workers, whereas musicians are indoor workers. For example, musicians probably would have had significant occupational exposure to passive smoking in the 1970s, 1980s and 1990s prior to Australian legislation to ban indoor smoking in the early 2000s [8].

There have been no published studies to date analysing the life expectancy of Australian popular musicians. Studies looking at life expectancy of popular musicians in the US [9], UK [10] and Europe [11] have all found that these musicians tend to die earlier (at higher rates) than the general population.

The primary aim of this study was to analyse the death rates of Australian popular (including rock) musicians and Australian athletes (namely male AFL footballers and male cricketers) and to compare these to (expected) death rates of the general population. Secondary aims included to make disease-specific comparisons and to calculate survival trajectory and life expectancy from age 20 for the three groups.

## 2 Methods

We used Wikipedia output to create our cohorts for study, as Wikipedia is now considered to be the most comprehensive database on lifespan of athletes and similar celebrities [[12], [13], [14]]. We tried to replicate methods that were used for previously published analyses of male AFL/VFL footballers and male Australian cricketers [5,7]. Both of these studies used cohorts of athletes starting in the year 1971 until the most recent year of published data. Male-only cohorts were used for these athlete groups as females have only become sufficiently prominent in these sports in the 21st Century, hence with very low expected death rates due to young age.

For the current study, the AFL dataset was updated to include cohort additions and deaths in 2021 and 2022. The only major change in methods used for the footballer cohort is that we used Australian (rather than Victorian) male death rates for 1971–2022 to maintain consistency with the cricket and musician methodology. The cohort used for the cricket analysis was updated to include 2022 deaths.

A dataset for Australian rock and popular musicians was created by:

(1) Searching Wikipedia for pages in the categories “Australian rock musicians” and “Australian pop musicians” on July 20, 2023;
(2) Excluding bands, groups and duos, although individuals within a group were kept if they had their own individual page;
(3) Excluding any musician who had died before 1971; and
(4) Using the data in Wikipedia pages as the source of information for year of birth, year of debut as a popular performer or recording artist and year of death.

Where year of birth or year of debut were unavailable, estimates were made in the following order, noting that the average age at professional music “debut”, where this was available, was 20.35 years:

- Google search for the name(s) of the recording artist and “How old is ….”?
  ○ If year of birth could be estimated from this search, it was used.
- If year of debut was known but year of birth was unknown, it was presumed that debut occurred at age 20, and Year of Birth was estimated as Year of Debut minus 20.
- If Year of Birth was known or estimated from the Google search but Year of Debut was unknown, then Year of Debut was estimated as Year of Birth plus 20.
- All artists were presumed to be “Australian” if they were categorized as Australian rock or pop musicians even if they had dual nationality.
- Where the musician had died, cause of death (if available, including if listed on the Wikipedia page or one of the references) was recorded.

The updated Australian rates of death (separately for males and females, and by five-year age group and year, and by all deaths combined and only cancer deaths) were obtained from the Australian Bureau of Statistics [15].

Standardized Mortality Ratios (SMRs) were calculated as follows. The number of Observed deaths for an age-sex-year group was calculated directly based on the information from the Wikipedia pages. The Expected number of deaths was calculated by multiplying the age-sex-year-specific number in each cohort by the relevant age-sex-specific 5-year mortality rate in the Australian population. The SMR was calculated by dividing Observed by Expected. Overall SMRs were calculated by first summing across all ages for each year and decade. Ninety-five per cent confidence intervals were calculated using the “epitools” package in R software (version 4.4.0, GNU project) [16].

The majority of musicians had cause of death listed on their Wikipedia page although the majority of athletes did not. An additional SMR was calculated for death from cancer in musicians.

The expected percentage of deaths due to cancer (for this musicians group) was calculated specific to each decade and 10-year age cohort. By decade of life, the lowest percentage of (expected) deaths due to cancer was 8% (in the 20–29 year old age group) and the highest percentage of expected deaths was 45% of all deaths being due to cancer (in 50–69 year olds from the 1990s onwards) [17].

Median survival time for the various groups in the study (for a given decade) was assessed by calculating the age at which >50% of the reference population would still be alive. For the general population this was done starting at age 20 (assuming 100% survival through infancy, childhood and teenage years) to be a fair comparison with the musician and athlete groups. Curves which were plotted for the general population showing survival rates over time were calculated for our study cohorts using the calculated decade-by-decade Standarised Mortality Ratios for the cohorts compared to the general population.

The study was considered to be exempt from requiring Ethics Committee approval as it used only publicly-available data.

## 3 Results

For the musicians dataset, there were 897 Wikipedia pages covering one of the two categories (after excluding 38 duplicate pages). Further exclusions were pages focused on bands, groups or duos. One of the solo artists was excluded as she died in 1968 (the only record of an Australian rock or pop solo musician from this cohort dying before 1971). This left 222 female and 433 male solo artists (a total of 655) in the overall musicians cohort for this study. Seventy-seven of these musicians had died by the end of 2022 and 571 were still alive (Table 1).

**Table 1:** Cohort characteristics.

	AFL/VFL footballers	Sheffield Shield cricketers	Rock & pop musicians	Male musicians	Female musicians
Cohort size	10,022	1817	655	433	222
Sex	Male	Male	Combined	Male	Female
Person years of follow-up	294,049	54,253	19,111	14,221	4890
Deaths in cohort	4048	600	77	63	14

The cricketers and footballers had reductions in SMRs compared to the general Australian population (Table 2). Popular musicians had an estimated 35% higher risk of death than the general population (Table 2). Female musicians had a higher estimated SMR than males.

**Table 2:** Standardized mortality ratios (SMRs) for cohorts versus expected rates for the general Australian population (of the same sex).

	AFL/VFL footballers	Sheffield Shield cricketers	Rock & pop musicians	Male musicians	Female musicians	Cancer deaths in musicians
Expected deaths	5262	848	57.0	49.6	7.4	19.4
Actual deaths	4048	600	77	63	14	36
SMR ​± ​95% CI	0.77 (0.74–0.80)	0.71 (0.64–0.78)	1.35 (1.07–1.71)	1.27 (0.97–1.65)	1.90 (1.11–3.26)	1.85 (1.32–2.59)

The causes of death of the musicians are listed in Table 3, showing a large preponderance of cancer deaths (almost half of all deaths, and more than half where the cause was known). The primary cancers were from a variety of body sites. Cause-specific analysis for cancer was performed, showing an increase of expected deaths due to cancer in musicians - SMR 1.85 (95% CI 1.32–2.59). There were not enough individual deaths to undertake diagnosis-specific statistical analysis, but Table 3 reveals the exact diagnoses.

**Table 3:** Listed causes of death for Australian rock and pop musicians.

Cause (general)	Specific	Number of deaths	Ages of death
Cancer		36	
	Liver	5	50,54,55,61,63
	Brain	5	49,54,57,58,67
	Prostate	3	59,60,65
	Head & neck (throat)	3	45,52,63
	Lung	2	46,77
	Breast	2	54,74
	Myeloma	2	55,68
	Pancreatic	2	43,54
	Bowel	1	53
	HIV-related	1	48
	Oesophagus	1	64
	Melanoma	1	67
	Leukaemia	1	50
	Lymphoma	1	60
	Not-specified	6	37,42,62,66,66,77
Cardiovascular		10	
	Cardiac arrest	5	46,48,48,61,64
	Heart disease	2	60,68
	Stroke	2	47,72
	Aorta	1	67
Injury/violence		14	
	Suicide	6	36,46,51,54,58,68
	Homicide	1	39
	Air crash	1	49
	Drug overdose	3	34,37,43
	Alcohol related	2	34,62
	Fall	1	72
Other		17	
	Dementia	2	64,79
	Kidney disease	2	57,77
	Lung disease	2	69,79
	Pneumonia	2	66,68
	Seizure	1	29
	Illness	1	70
	Unknown	7	66,70,71,72,75,75,88

There were a moderate number of deaths related to injuries, overdoses and other violent causes (20% of all deaths) (Table 3).

Table 4 shows the ages of deaths of the male musicians versus the athletes and the expected versus actual numbers for each decade of life. The athletes had lower than expected rates of death in all decades, but there was a gradual rise (becoming closer to the expected rate) as they became older. This trend was very similar in the football and cricket cohorts (Table 4(b)). Musicians appeared to suffer higher numbers of deaths than expected in the decades of their 40s, 50s and 60s, although small sample size limits the confidence in this finding.

**Table 4:** (a) and (b) – Decades of deaths of Australian popular musicians versus athletes.

	Male musicians		
Decade	Actual	Expected	SMR
<40	5	9.2	0.54 (0.23–1.31)
40s	13	6.4	2.03 (1.17–3.54)
50s	14	9.2	1.52 (0.89–2.61)
60s	22	12.1	1.82 (1.17–2.84)
70s	8	8.9	0.90 (0.43–1.87)
>79	1	3.9	0.26 (0.03–1.96)
	63	49.6	

	Male footballers			Male Cricketers		
Decade	Actual	Expected	SMR	Actual	Expected	SMR
<40	40	111.9	0.36 (0.26–0.49)	4	21.6	0.19 (0.07–0.49)
40s	60	137.4	0.44 (0.34–0.56)	9	26.4	0.34 (0.18–0.66)
50s	218	355.5	0.61 (0.54–0.70)	35	62.0	0.56 (0.40–0.79)
60s	576	848.5	0.68 (0.62–0.74)	82	138.3	0.59 (0.47–0.74)
70s	1182	1529.4	0.77 (0.73–0.82)	160	234.3	0.68 (0.58–0.81)
>79	1971	2279.3	0.86 (0.82–0.92)	310	365.8	0.85 (0.73–0.98)

Table 5 shows the mean survival age by decade for the various groups considered in this study. The general population groups were calculated starting at age 20 (assuming 100% survival to age 20 to match debut age for musicians and athletes). The SMRs in Table 4 (a) and (b) were used to adjust general male population death rates for the various groups at each age decade. No calculations were made for female musicians due to the small sample size and small number of deaths. For all groups, life expectancy improved from decade to decade over time and the male general population had a shorter life expectancy than the female general population (with the gap reducing slightly over the five decades).

**Table 5:** Median survival age for the groups analysed (by decade).

	Male general population	Male footballers	Male cricketers	Male musicians	Female general population
1971–80	72	76	77	66	79
1981–90	74	78	79	70	81
1991–00	77	80	81	75	83
2001–10	80	83	84	79	85
2011–22	83	85	85	82	86

**Fig. 1:**
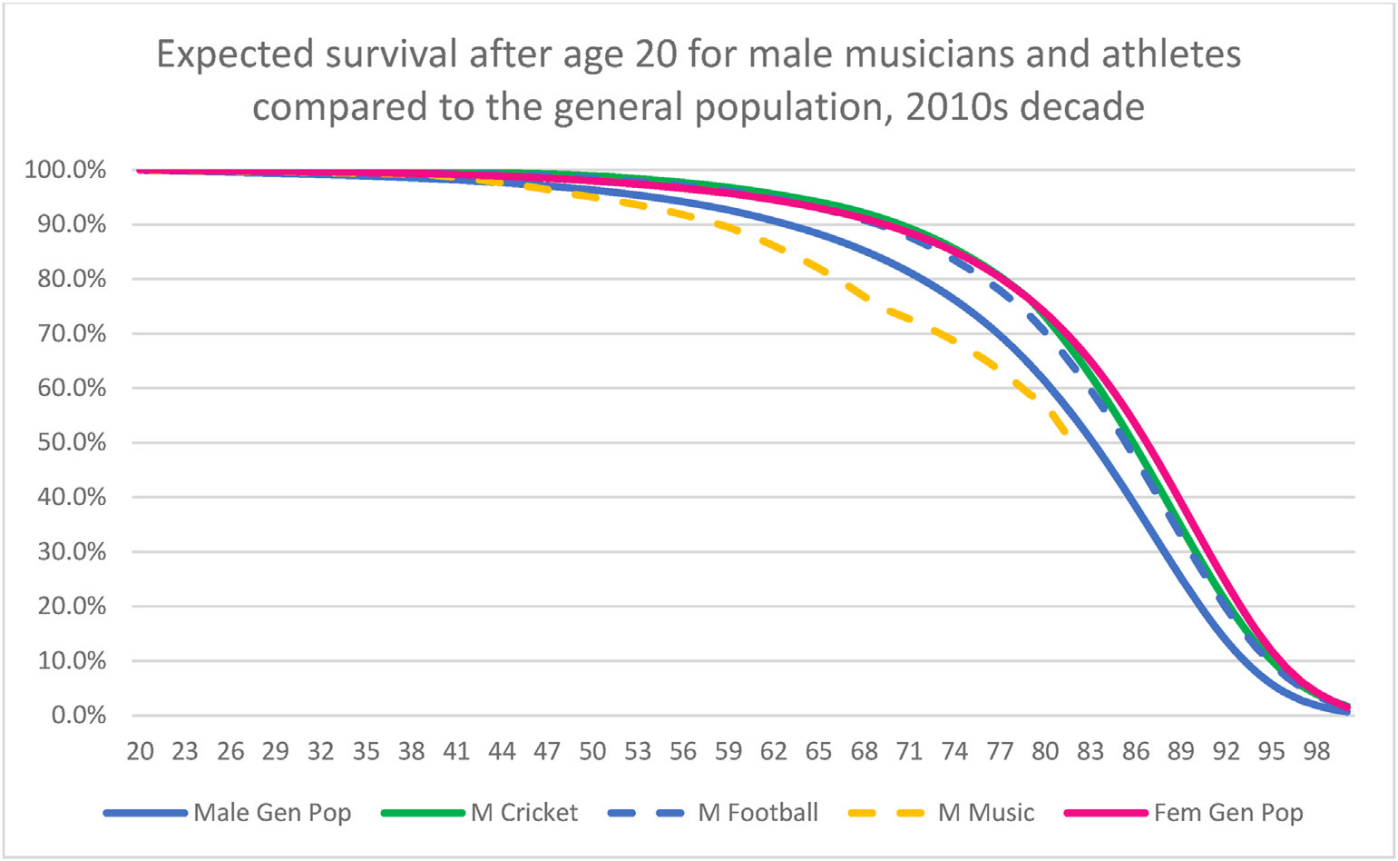
Expected survival curve from age 20 onwards for male athletes and musicians compared to the general population for the decade of the 2010s.

Figure 1 shows the expected survival (in the decade of 2010s) for the three male subject cohorts compared to the male and female Australian general population, using the same methodology as Table 5 (based on the SMRs in Table 4). This visualises the relative survival benefit of the male athlete groups and the negative effect seen in middle-age in the male musician group. Life expectancy and survival for the male athlete groups from age 20 onwards roughly tracked the superior life expectancy of the general Australian female population until the age of 70 (football cohort) and the age of 80 (cricket cohort). Life expectancy for the male musician group was worse than the general male population in middle age.

A supplemental Excel file is included to demonstrate calculations made in this study and to list the musicians and athletes in the various cohorts (from publicly-available sources).

## 4 Discussion

This is the first analysis of SMRs of Australian rock and popular musicians. The findings suggest these musicians have a marginally-increased risk of death overall and from cancer in middle-age specifically compared to the general population. Our confidence that the findings are true associations improves due to consistency with previously-published international studies. The higher death rates for the musician groups is in contrast to reduced death rates for the two athlete groups (footballers and cricketers).

There were few deaths at performing age (20s and 30s) for Australian musicians – lower than the general population. There were a higher number than expected deaths in 40s, 50s and 60s, and particularly for deaths from cancer. No single cancer type was particularly prominent, but some of the cancers (e.g. gastrointestinal or respiratory tract) could relate to substance exposure and perhaps explain any excess deaths observed in this cohort. Of the 77 dead musicians listed on Wikipedia, twelve of the pages described a publicly-recorded history of illicit drug or substance abuse. Being indoor workers, musicians working in the 1960s, 1970s, 1980s or 1990s were likely to have been subjected to passive smoking in the workplace, with a high percentage also perhaps being actual smokers.

Cancer related to substance use (alcohol, smoking and perhaps illicit drug use) can be suspected from some of the locations (throat, lung, liver, bowel, pancreas, oesophagus). A previous review of substance abuse and cancer risk concluded that there was sufficient evidence to assume illicit substances increased the risk of suffering many types of cancer [18], although the overlap between illicit substance use and legal substance exposure (alcohol and tobacco) makes it hard to ascribe a particular cause. Furthermore, some cancer types seen in this musicians cohort (such as brain and haematological) are probably not related to lifestyle factors.

Musicians in this Australian cohort did not exhibit an excess of deaths at very young ages. This is in contrast to US and UK popular musicians, where death at young age is a well-documented phenomenon. There is a famed “27 club” of popular musicians who have died at this specific age, including Amy Winehouse, Kurt Cobain, Jimi Hendrix, Jim Morrison, Janis Joplin and Brian Jones [10]. A study of a British cohort of popular musicians failed to uncover evidence of the age of 27 being particularly cursed, but did find that musicians died at a rate of two to three times the general population throughout their 20s and 30s [10]. We found neither a single Australian musician member of the “27 Club” nor an excess of deaths in 20s and 30s in Australian rock and pop musicians.

A US cohort with far-greater numbers than this study showed lifelong increases in death rates of over 10,000 popular musicians compared to the general US population [9]. There were significant increases in all of the categories of accidents, drug overdoses, suicide and homicide. Cancer deaths were also increased, but not to the same relative extent of the increased risk of the so-called violent deaths. Because cancer is one of the most common causes of death, the absolute increase may have been larger. In this large US cohort, 25–27% of rock and pop musicians died from cancer, whereas in the general US population about 17% of people die of cancer [9].

A similar study which included European musicians found both US and European musicians had significantly higher death rates than the reference populations [11]. The study estimated that 25% of the premature deaths related to chronic drug or alcohol problems, although it is not clear whether this related to direct cause of death (such as a drug overdose) or indirect (such as head and neck or liver cancer).

There were some slight differences in methods between the musicians cohort and the sporting cohorts. For the sporting cohorts, dates of birth, debut and death were reliably recorded, either on Wikipedia or another reliable source (almost for every athlete). With respect to the Wikipedia pages for musicians, year of death was very reliably reported, year of birth was generally reported but year of musical “debut” was inconsistently reported. First match (in the VFL/AFL or Sheffield Shield) is tightly defined for the athletes but a more nebulous concept for musicians, as there is a gradual increment from public performances, recording of singles and albums and then international touring. By comparison with the athlete cohorts on Wikipedia, if a musician had died the cause of death was much more consistently recorded. The musicians cohort was also younger than the athlete cohort, because rock and pop musicians only emerged in the 1960s in Australia, whereas the VFL football and Sheffield Shield cricket groups were forming from as early as the 1890s onwards.

We consider that our athlete cohorts exhibited a “Healthy Worker Hire Effect” which would be expected to reduce early deaths in them (but with the percentage effect unspecified). We did not consider that the athlete cohorts had any “Healthy Worker Survivor Effect” (HWSE) as the cohorts were sharply defined on debut game (VFL/AFL match or Sheffield Shield match). By contrast, the HWSE was likely to also be a factor in the musicians. A reference covering Australian rock music [19] which was written in 1988 (well before the first Wikipedia page was created in 2001) had a “Dedication” page to 28 “Australian rock and pop stars who are no longer with us”, who died between 1962 and 1988. Only 5 of these 28 have Wikipedia pages in the Australian rock or pop musicians category (and the vast majority do not have a Wikipedia page at all). Of the 23 deaths not in our cohort, all had a cause of death listed, with the most common causes being cancer (5), car accident (5), cardiac (5) and drug overdose (3). The number of musicians who worked during the period is not known; nor is it known how many more of them may have become famous enough to have been worthy of a Wikipedia page if they had survived. Being less well-defined cohorts than the athlete cohorts, slightly more caution should be added to our findings on musicians. As our results are consistent with the published international studies on popular musician longevity, we consider the observation that musicians have excess deaths in middle-age likely to be a true finding.

The study of Kenny et al. [9] found that musicians from earlier genres (classical, jazz, blues, country) and who lived predominantly in the early 20th Century did not die earlier than the general population. The genres that became popularized in the 1960s onwards (rock and pop from the 1960s, then later rap and hip-hop) have exhibited higher age-adjusted rates of death than the general population. These observations suggest that the lifestyle association of substance abuse which has been part of the popular music culture since the 1960s may well be the underlying cause of premature deaths in modern-era musicians. However, other explanations are possible, such as occupational exposures (e.g. passive smoking).

In our cohort, we observed excess cancer deaths in the 5th, 6th and 7th decades of life for popular musicians. In other musical cohorts, drug overdose deaths were observed earlier in life. It is possible we did not find this in our cohort because of the HWSE in developing the Wikipedia cohort. Our Australian study was consistent with the work of Kenny et al. relating to US musicians in showing that being female was not protective from early death in popular musicians [20].

The identified deaths in the musicians in this study are biased towards famous musicians from the mid to late 20th Century. It is possible that in this era there was a more reckless alcohol, tobacco and drug taking culture in musicians which may not necessarily reflect behaviour of musicians in the 21st Century.

The definition of the cohort of Australian rock and pop musicians on Wikipedia (i.e. the category is a judgment call of Wikipedia editors) is a limitation. There are clearly some musicians who are Australian and in the rock or pop genre who are only listed in the category “Australian musicians”. However, trying to include these in the cohort would have introduced a considerable bias into our study, because it would be difficult to avoid significant subjectivity in deciding who of the performers in the “Australian musicians” Wikipedia category should also be considered as rock and pop musicians rather than as other types of musicians. An example of the incomplete cohort, the band Rose Tattoo has previously announced that five band members have died of cancer (Dallas Royall (1991), Peter Wells (2006), Ian Rilen (2006), Lobby Loyde (2007) and Mick Cocks (2009)). Three of these band members (Royall, Wells and Cocks) have Wikipedia pages in the Australian rock musicians category and hence were part of this study cohort, whereas Rilen and Loyde did not have Wikipedia pages and so were not included in the cohort. The cohort is therefore probably incomplete, but the advantage of the approach used is that it is likely almost all included performers genuinely belonged in the cohort.

When the Australian sporting cohorts are compared to the musical cohort, the likelihood that the observed improvement in longevity for the athletes is a “real” phenomenon increases. We previously concluded that reduced death rates in athletes were either due to the health benefits of exercise (real effect of the occupation) or the HWHE (artefact from the selected cohort). Musicians must be physically fit but not to the same extent as athletes, and they work indoors rather than outdoors. Musicians also may continue to work into their 40s whereas by this age nearly all footballers and cricketers have retired from professional sport. Finally, whereas the associated lifestyle for musicians may predispose them to cancers (one of the most common causes of death), the lifestyle of athletes (who exercise to a high level) almost certainly reduces their risk of cancer and cardiovascular disease. Recently, 13 cancers have been shown to have physical inactivity as a risk factor in the Australian population [21], consistent with international studies [2,[22], [23], [24]].

The practical implications of these results are not straightforward for Australian rock and pop musicians, who have a far less formal medical support network than athletes. It is unlikely that cancer screening beyond that recommended for the general population is warranted. However, it would be appropriate to advise older musicians of their probable increased risk of various cancers and that it would be sensible to stay vigilant for early signs of these and to have regular contact with their general practitioner. For younger musicians, general advice regarding avoiding tobacco smoking, excess consumption of alcohol and illicit drugs for health reasons is sufficient.

## 5 Conclusions

In conclusion, this study has made findings consistent with international studies showing that athletes outlive the general population but that this survival benefit is not seen in famous musicians. This is the first Australian-specific study of rock and pop musicians. We found a higher rate of cancer death in middle age. Lifestyle factors (alcohol, tobacco and possibly other drug consumption) seem the most likely explanation for this finding, but this study could not assess this specifically or rule out other exposures as being contributory. Athletes are likely to have survival benefits associated with being more regular exercisers than the general population, with exercise known to protect against cardiovascular disease and cancer.

## Declaration of competing interest

John W. Orchard is Chief Medical Officer for Cricket Australia (with respect to the manuscript itself).

Jessica J. Orchard is EIC for JSAMS Plus [and was excluded from any role in the review process].

## References

[1] Antero J., Tanaka H., De Larochelambert Q., Pohar-Perme M., Toussaint J.F. (2021). Female and male US Olympic athletes live 5 years longer than their general population counterparts: a study of 8124 former US Olympians. Br J Sports Med.

[2] Antero-Jacquemin J., Pohar-Perme M., Rey G., Toussaint J.F., Latouche A. (2018). The heart of the matter: years-saved from cardiovascular and cancer deaths in an elite athlete cohort with over a century of follow-up. Eur J Epidemiol.

[3] Lemez S., Baker J. (2015). Do elite athletes live longer? A systematic review of mortality and longevity in elite athletes. Sports medicine - open.

[4] Ehrlich J., Kmush B., Walia B., Sanders S. (2019). Mortality risk factors among National Football League players: an analysis using player career data. F1000Research.

[5] Orchard J.W., Orchard J.J., Semsarian C., La Gerche A., Driscoll T. (2022). Reduced death rates of elite Australian Rules footballers compared to age-matched general population. J Sci Med Sport.

[6] Clarke P.M., Walter S.J., Hayen A., Mallon W.J., Heijmans J., Studdert D.M. (2012). Survival of the fittest: retrospective cohort study of the longevity of Olympic medallists in the modern era. Bmj.

[7] Luies N., Orchard J.J., Driscoll T., Sahdra S.K., Cheng J., Davis A. (2023). Sheffield Shield cricketers live longer than the age-matched general Australian male population. Indian J Orthop.

[8] Grace C., Smith L., Greenhalgh E., Scollo M., Winstanley M. (2021). Tobacco in Australia: Facts and issues.

[9] Kenny D.T., Asher A. (2016). Life expectancy and cause of death in popular musicians: is the popular musician lifestyle the road to ruin?. Med Probl Perform Art.

[10] Wolkewitz M., Allignol A., Graves N., Barnett A.G. (2011). Is 27 really a dangerous age for famous musicians? Retrospective cohort study. BMJ.

[11] Bellis M.A., Hennell T., Lushey C., Hughes K., Tocque K., Ashton J.R. (2007). Elvis to Eminem: quantifying the price of fame through early mortality of European and North American rock and pop stars. J Epidemiol Commun Health.

[12] Kovbasiuk A., Ciechanowski L., Jemielniak D. (2024). A taste of ambrosia: do Olympic medalists live longer than Olympic losers?. Scand J Publ Health.

[13] Mannix L. (2022 Sept 13). Evidence suggests wikipedia is accurate and reliable. When are we going to start taking it seriously?. Syd Morning Her.

[14] Harrison S. Who updates celebrity deaths on wikipedia? *Slate*2018. https://slate.com/technology/2018/2008/the-people-who-update-wikipedia-pages-when-celebrities-like-aretha-franklin-die.html.

[15] ABS (2023). Deaths, year of registration, age at death, age-specific death rates, sex, states, territories and Australia. https://explore.data.abs.gov.au/vis?tm=deaths&pg=0&hc%5bMeasure%5d=Deaths&df%5bds%5d=PEOPLE_TOPICS&df%5bid%5d=DEATHS_AGESPECIFIC_REGISTRATIONYEAR&df%5bag%5d=ABS&df%5bvs%5d=1.0.0&pd=%2C&dq=12.1.A99%2BA95%2BA90%2BA85%2BA80%2BA75%2BA70%2BA65%2BA60%2BA55%2BA50%2BA.

[16] Aragon T.J., Fay M.P., Wollschlaeger D., Omidpanah A. (2020). https://CRAN.R-project.org/package=epitools.

[17] AIHW Cancer in Australia. https://www.aihw.gov.au/reports/cancer/cancer-data-in-australia/contents/overview-of-cancer-in-australia-2023.

[18] Moussas G.I., Papadopoulou A.G. (2017). Substance abuse and cancer. Psychiatriki.

[19] McGrath N. (1988).

[20] Kenny D.T., Asher A. (2017). Gender differences in mortality and morbidity patterns in popular musicians across the lifespan. Med Probl Perform Art.

[21] Ellis L., Milne R.L., Moore M.M., Bigby K.J., Sinclair C., Brenner D.R. (2024). Estimating cancers attributable to physical inactivity in Australia. J Sci Med Sport.

[22] Ding D., Van Buskirk J., Nguyen B., Stamatakis E., Elbarbary M., Veronese N. (2022). Physical activity, diet quality and all-cause cardiovascular disease and cancer mortality: a prospective study of 346 627 UK Biobank participants. Br J Sports Med.

[23] Garcia L., Pearce M., Abbas A., Mok A., Strain T., Ali S. (2023). Non-occupational physical activity and risk of cardiovascular disease, cancer and mortality outcomes: a dose-response meta-analysis of large prospective studies. Br J Sports Med.

[24] Moore S.C., Lee I.M., Weiderpass E., Campbell P.T., Sampson J.N., Kitahara C.M. (2016). Association of leisure-time physical activity with risk of 26 types of cancer in 1.44 million adults. JAMA Intern Med.

